# Emerging vector-borne diseases in dromedaries in Tunisia: West Nile, bluetongue, epizootic haemorrhagic disease and Rift Valley fever

**DOI:** 10.4102/ojvr.v84i1.1316

**Published:** 2017-03-31

**Authors:** Thameur B. Hassine, Jihane Amdouni, Federica Monaco, Giovanni Savini, Soufien Sghaier, Imed B. Selimen, Walid Chandoul, Khaled B. Hamida, Salah Hammami

**Affiliations:** 1 Ecole Nationale de Médecine Vétérinaire de Sidi Thabet, Université la Manouba, Tunisie; 2 Université Tunis El Manar, Institut de la Recherche Vétérinaire de Tunisie, Tunisie; 3 Istituto Zooprofilattico Sperimentale dell’Abruzzo e del Molise, Teramo, Italy; 4 Commissariats Régionaux au Développement Agricole, Medenine, Tunisia

## Abstract

A total of 118 sera were collected during 2016 from two groups of dromedaries from Kebili and Medenine governorates in the south of Tunisia. The aim of this study was to provide the first serological investigation of four emerging vector-borne diseases in two groups of dromedaries in Tunisia. Sera were tested by ELISA and serum neutralisation test to identify West Nile virus (WNV), bluetongue virus (BTV), epizootic haemorrhagic disease virus (EHDV) and Rift Valley fever virus (RVFV). In the first group, the seroprevalence for BTV was 4.6%, while in the second group, it was 25.8% for WNV and 9.7% for BTV. Only serotype 1 was detected for BTV in the two groups. No evidence for circulation of RVF and EHD viruses was revealed. Results indicated that dromedaries can be infected with BTV and WNV, suggesting that this species might play a significant role in the epizootiology of these viral diseases in Tunisia and neighbouring countries.

## Introduction

The economic value of the dromedary as a source of meat and transportation with the renewed interest around the Middle East Respiratory Syndrome Coronavirus (MERS-CoV) in the Arabian Peninsula raises the question of the impact of the dromedary on new and emergent diseases in North Africa.

The effective control of West Nile (WN), bluetongue (BT), epizootic haemorrhagic disease (EHD) and Rift Valley fever (RVF) depends on a thorough understanding of the possible implications of dromedaries in the replication or the dissemination of the West Nile virus (WNV), bluetongue virus (BTV), epizootic haemorrhagic disease virus (EHDV) and Rift Valley fever virus (RVFV).

Tunisia is located at the interface between the sub-Saharan region and Europe. It has encountered several episodes of emerging vector-borne diseases such as WN, BT and EHD. Tunisia has experienced three major WN epidemics that have particularly affected humans in 1997, 2003 and 2012 (Ben Hassine et al. 2015). In 2015, an equine with clinical signs was reported for the first time in the south of Tunisia in the oases of Tozeur (OIE 2015). Since the first occurrence of BT in 1999, outbreaks have been reported in Tunisia and three serotypes, namely BTV2, BTV1 and BTV4 were identified in 2000, 2006 and 2009, respectively (Hammami 2004; Sghaier et al. 2014). More recently, other BT outbreaks because of serotype 1 in 2011 and serotype 4 in 2013 have been reported (Lorusso et al. 2013; Sghaier et al. 2014). In 2015, a study suggested an active circulation of RVFV and evidence of human exposure in the population of Tunisia was reported (Bosworth et al. 2016). EHDV was detected for the first time in Tunisia in 2006. Recently, clinical cases in cattle were reported in countries surrounding the Mediterranean Basin, including Morocco, Algeria and Tunisia, with the emergence of EHDV serotype 6 (Ben Dhaou et al. 2016). The aim of the present study was to provide the first serological investigation of four emerging vector-borne diseases in two groups of dromedaries in Tunisia with a focus on WNV, BTV, EHDV and RVFV.

## Materials and methods

**FIGURE 1:**
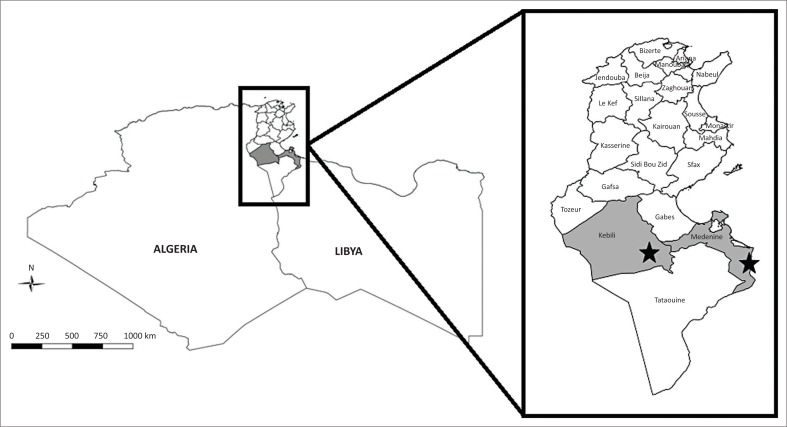
Geographical location of sampled dromedaries in the south of Tunisia (Kebili and Medenine governorates).

This study was carried out in 2016 in the governorates of Kebili and Medenine (Figure 1). These two governorates were selected because of their relatively high dromedary density and cross-border animal movement within the region (Ayari-Fakhfakh et al. 2011). These factors represent high-risk zones for disease transmission in Tunisia.

The first lot of 87 sera was collected from dromedaries intercepted at the country border posts by the Tunisian veterinary services in the region of Ben Guerden (governorate of Medenine) where the dromedaries usually circulate in the arid desert. The second lot of 31 sera was collected from individual dromedaries in contact with sheep herds in the oases of Kebili, which are characterised by traditional irrigation where the main practice is flooding (Mekki et al. 2013). Sera were tested by enzyme-linked immunosorbent assay (ELISA) for WNV, BTV, RVFV and EHDV. IgG antibodies to RVFV were detected using ID Screen^®^ RVF competition multi-species ELISA kits (ID Vet; Innovative Diagnostics, Montpellier, France). For EHD, all the sera were tested by a blocking competitive ELISA (LSI VET EHDV Blocking, LSI, France) to detect the specific anti-EHDV VP7 antibodies. Sera were screened for the presence of group-specific BTV antibodies by using the c-ELISA IDEXX Bluetongue Competition^®^ assay (IDEXX BT, Hoofddorp, the Netherlands). The Usutu virus (USUV)- and WNV-specific antibodies were detected using a commercially available competition ELISA, (ID Screen^®^ West Nile Competition, ID Vet, Montpellier, France). Serum neutralisation (SN) test for 26 BTV serotypes and for WNV–USUV was used on positive samples (Di Gennaro et al. 2014). The test was conducted at the Istituto Zooprofalitico Sperimentale dell’Abruzzo e del Molise ‘G. Caporale’ (IZSAM) and OIE reference laboratory for BTV and WND, under biosafety level 3 conditions as previously described (OIE 2016).

## Results and discussion

Seropositivity of BTV was detected in the two groups (4.6% in the group of Medenine vs. 9.7% in the group of Kebili). This study confirms the circulation of serotype 1 in Tunisia that was detected for the last time in 2011 (Sghaier et al. 2014). For BTV, Tunisia has adopted a vaccination strategy in sheep using a bivalent vaccine (BTV1 and BTV4), but dromedaries are not vaccinated. Three species of *Culicoïdes* were predominantly detected in the south of Tunisia (Gabes governorate): *Culicoïdes jumineri, Culicoïdes sahariensis* and *Culicoïdes submarimitimus* although *Culicoïdes imicola* is considered the main vector of BTV (Mellor 1996; Sghaier et al. 2009). According to Batten et al. 2011, dromedaries seem to act as reservoirs, possibly playing a role in the spread of the disease by helping the virus to get through the geographic barrier which is represented by the Sahara desert. This desert stands between the tropics and subtropics, where BTV is endemic, and North Africa, where it periodically causes epizootics. However, there is scarce information about the clinical manifestations of BT in dromedaries. South American camels are susceptible to BTV infection, but they develop only a mild form of the disease (Schulz et al. 2012). For WNV, this study revealed that 25.8% of the dromedaries, which live in oases, were seropositive. This result is consistent with the findings reported by Ben Hassine et al. (2015), where a high seropositivity in horses was found in the oases of Kebili that were identified as high-risk areas for WNV circulation in Tunisia. Moreover, the presence of *Culex pipiens* in the south of Tunisia and isolation of WN virus from *C. pipiens* in central Tunisia (lineage 1) confirms this finding (Wasfi et al. 2016). Recent studies in North Africa reported WNV seropositivity rates in dromedaries ranging from 13% (Touil et al. 2012) to 29% (El-Harrak et al. 2011). Similar to human and horse populations, in which less than 1% of WNV-infected individuals become severely ill, it appears that the majority of camels infected with WNV are asymptomatic and recover uneventfully. No evidence for the circulation of RVF and EHD viruses are revealed in this study. Serologic evidence of RVF in dromedaries is frequently reported (Swai & Sindato 2015), yet the description of clinical signs is rare. Subclinical, mild forms and healthy carriers of the virus (Paweska 2015; Swanepoel & Coetzer 2004) have been reported. The presence of the RVF competent vector *C. pipiens* and *Aedes caspius* in oases make this ecosystem favourable for RVF transmission in Tunisia where *Aedes* could be responsible for the initiation of an outbreak and *Culex* maintains the virus activity (Soti et al. 2012). For EHDV, dromedaries seem not to be involved in the disease transmission. The study conducted by Wernery et al. (2013) using ELISA test showed that 29% of dromedaries from the United Arab Emirates have antibodies to EHDV.

## Conclusion

This study, for the first time, reports infection of dromedaries with WNV and BTV in Tunisia, confirming the reported infections of dromedaries with BTV and WNV in North Africa and the Middle East. A larger serosurvey with an entomological monitoring and a syndromic surveillance in a One Health concept is needed to understand the implications of dromedaries in the transmission of emerging arthropod-borne viruses which can be very useful for the purposes of control and planning surveillance activities not only in Tunisia but also for all the regions of North Africa.
